# Cognitive impairment after intravenous thrombolysis in mild stroke: assessment of cerebral blood flow covariance network

**DOI:** 10.3389/fneur.2025.1513182

**Published:** 2025-03-07

**Authors:** Kefu Mei, Feng Li, Zhiming Kang, Dong Sun, Xuefei Luo, Shiyuan Tian, Lei Zhang, Junjian Zhang

**Affiliations:** ^1^Department of Neurology, Zhongnan Hospital of Wuhan University, Wuhan, China; ^2^Department of Neurology, Xiangyang Central Hospital, Affiliated Hospital of Hubei University of Arts and Science, Xiangyang, China; ^3^Department of Radiology, Xiangyang Central Hospital, Affiliated Hospital of Hubei University of Arts and Science, Xiangyang, China; ^4^Xiangyang Polytechnic, Xiangyang, China

**Keywords:** mild stroke, intravenous thrombolysis, cognitive impairment, cerebral blood flow, covariance network

## Abstract

**Background:**

Mild stroke may lead to cognitive impairment, and it remains unclear whether intravenous thrombolysis (IVT) can mitigate cognitive deficits. This study investigates whether IVT can help alleviate cognitive function impairment in patients and further explores changes in the topological properties of cerebral blood flow (CBF) networks.

**Methods:**

This observational study prospectively enrolled 94 patients with acute mild ischemic stroke (44 IVT vs. 50 non-IVT) from two hospitals. A battery of neuropsychological tests and arterial spin labeling were performed to evaluate their cognitive functioning and CBF in 116 brain regions. Voxel-wise CBF was compared between patients and health controls. The CBF covariance network of patients was constructed by calculating across-subject CBF covariance among 116 brain regions. Network properties were calculated and compared between IVT and no-IVT groups.

**Results:**

The mild stroke group demonstrated significantly lower Montreal Cognitive Assessment (MoCA) scores compared to healthy controls (*p* < 0.001). Patients receiving IVT showed superior performance on the Trail Making Test-B (*p* = 0.043), Clock Drawing Test (*p* = 0.001), and Verbal Fluency Test (*p* = 0.033). Multivariate regression analysis adjusted for covariates demonstrated significant associations between IVT and cognitive outcomes: Montreal Cognitive Assessment (*β* = 2.85; 95% CI, 0.64–5.13), Trail Making Test-A (*β* = −16.90; 95% CI, −32.89–-0.90), Trail Making Test-B (*β* = −43.27; 95% CI, −78.78–-7.76), Hopkins Verbal Learning Test-Revised total recall (*β* = 3.57; 95% CI, 1.36–5.78), HVLT-R delayed recall (*β* = 1.53; 95% CI, 0.43–2.63), Clock Drawing Test (β = 7.09; 95% CI, 2.40–11.79), and Verbal Fluency Test (*β* = 3.00; 95% CI, 1.33–4.68). IVT patients exhibited higher small-worldness, clustering coefficient, and global efficiency of the network compared to non-IVT patients.

**Conclusion:**

Intravenous thrombolysis demonstrated early cognitive benefits across multiple domains in patients with mild stroke. Improvement in the brain CBF covariance network properties may be the underlying mechanism.

## Introduction

Mild ischemic stroke, defined by a National Institutes of Health Stroke Scale (NIHSS) score of less than 6, constitutes over half of all ischemic stroke. While patients typically experience only mild disabilities, they still face significant long-term effects, including fatigue, cognitive impairment, anxiety, and depression (1, 2). The incidence of cognitive impairment following mild ischemic stroke can reach up to 60% within one year, yet no specific treatments currently exist (3, 4).

Intravenous thrombolysis (IVT) is the primary treatment for acute ischemic strokes within 4.5 h. National guidelines recommend prompt IVT within the therapeutic window for mild disabling strokes. However, the PRISMS trial (Study of the Efficacy and Safety of Alteplase in Participants with Mild Stroke) found no benefit of IVT for the mRS score in mild non-disabling stroke, leading to the exclusion of these cases from national recommendations (5). The lack of a clear definition differentiating disabling from non-disabling stroke complicates treatment decisions, as the effects of mild stroke extend beyond mRS assessments, leaving many clinicians hesitant to administer thrombolytic therapy (6).

Currently, there is limited research on the effects of IVT on cognitive impairment in patients with mild stroke. A small-sample study confirmed that alteplase can improve processing speed 90 days after onset (7). However, another observational study found no association between intravenous thrombolysis and MoCA scores (8). Post-stroke cognitive impairment significantly diminishes the quality of life for patients with mild stroke, emphasizing the need for further investigation of underlying mechanisms and new treatment strategies. Studies have identified abnormal CBF in patients experiencing cognitive decline (9, 10), highlighting issues such as reduced global gray matter CBF and altered blood flow in affected brain regions (11, 12). Recent advancements in brain network research, particularly using diffusion MRI and resting-state functional MRI, have enhanced our understanding of cognitive impairment at the connectome level. Arterial spin labeling (ASL) enables non-invasive measurement of brain blood flow, allowing CBF covariance network studies to effectively reflect changes in brain network parameters (13–15). However, the benefits of CBF network topological properties following intravenous thrombolysis in mild stroke remain unclear.

This study aims to explore whether IVT can mitigate cognitive function impairment and further investigate whether the protective role of the CBF covariance network is a potential mechanism. We hope that insights from these indicators will elucidate the potential benefits of intravenous thrombolysis for mild stroke and inform future clinical practices.

## Methods

### Subjects

From May 2023 to May 2024, we prospectively recruited 96 patients with mild ischemic stroke from Xiangyang Central Hospital and Wuhan University Zhongnan Hospital. Inclusion criteria were as follows: (1) Age 35–75 years; (2) First-time stroke, with normal cognitive function before onset (Informant Questionnaire on Cognitive Decline in the Elderly (IQCODE) > 64 points (15)); (3) Non-thrombolysis patients, extending the time from onset to admission to 12 h for individuals who did not receive emergency medical care via ambulance (16); (4) NIHSS score ≤ 5. Exclusion criteria included: (1) DWI-negative patients; (2) Unknown time of stroke onset; (3) Patients who underwent endovascular treatment; (4) A history of central nervous system diseases or injuries (e.g., brain tumors, inflammatory or infectious neurological diseases, traumatic brain injury with loss of consciousness, neurodegenerative diseases); (5) Inability to complete paper-and-pencil cognitive assessments (e.g., blindness); (6) MRI contraindications or poor image quality. Participants were contacted 24 h after onset to avoid interference or delayed treatment. They were further divided into IVT group or standard medicine group based on the treatment they received. Additionally, we selected healthy individuals matched for age, sex, and years of education from data from a study on risk factors for vascular cognitive impairment as the control group. This study was approved by the Ethics Committees of Wuhan University Zhongnan Hospital and Xiangyang Central Hospital affiliated with Hubei University of Arts and Science. Informed consent was obtained from all participants.

### Cognitive assessment

Each participant underwent a comprehensive battery of neuropsychological tests to assess global cognition and five key cognitive domains associated with vascular cognitive impairment: memory, language, attention, executive function, and visuospatial ability (see Table 1 for details). Testing was scheduled three to five days after the onset of symptoms, and the same experienced clinicians conducted all assessments. For all tests, except for the Trail Making Test (TMT), higher scores indicate better performance. TMT scores were converted to speed measures, calculated as 1/time (in seconds). Each test result was standardized to a normative score using z-scores.

**Table 1:** The battery of neuropsychological tests.

Cognitive domain	Screening instrument
Global cognition	Montreal Cognitive Assessment (MoCA)
Memory	Hopkins Verbal Learning Test (HVLT)
Attention	Trail Making Test-A (TMT-A)
Executive function	Trail Making Test-B (TMT-B)
Language	Verbal Fluency Test(VFT)
Visuospatial processing	Clock Drawing Test (CDT)

### MRI data acquisition

MRI data were acquired using two 3.0-Tesla scanners (Discovery MR750, GE Medical Systems, Milwaukee, WI; SIGNA Architect, GE Medical Systems, Milwaukee, WI). Foam padding was applied to stabilize the head and reduce movement. ASL perfusion imaging employed the pseudo-continuous ASL technique, utilizing numerous short pulses to mimic continuous labeling (TR/TE 5337/53.52 ms; number of excitation 3; flip angle 111^°^; post-label delay 2,500 ms; voxel resolution 1.875 × 1.875 × 4 mm; matrix, 128 × 128; scan time, 3:22 min). All images were visually inspected to ensure they were free of visible artifacts. MRI scans for all patients were scheduled within 48 h of symptom onset.

### MRI processing and connectivity analysis using graph theory

Pre-processing began with a visual quality assurance check of each CBF image, allowing for the removal of corrupted data. The CBF maps were normalized to the standard Montreal Neurological Institute (MNI) space using the following three steps: (1) the native ASL images from healthy controls were nonlinearly aligned to a standard perfusion template provided by SPM12 software (http://www.fil.ion.ucl.ac.uk/spm/software/spm12/) and averaged to create a study-specific ASL template; (2) all native ASL images were then nonlinearly normalized to this study-specific template; and (3) the CBF images were transformed to MNI space using the normalization parameters from step (2) and resampled to a voxel size of 2 × 2 × 2 mm^3^. To standardize the data, the CBF value at each voxel was divided by the whole brain’s mean CBF value.

To evaluate overall CBF changes in patients with mild stroke, we conducted a two-sample t-test on CBF maps derived from voxel-based analyses. Age, sex, and years of education were included as covariates to control for confounding in the regression analysis. Multiple comparisons were adjusted using the non-stationary cluster-level family-wise error (cFWE) method, with a significance threshold of *p* < 0.05.

A network consists of nodes and edges, where nodes represent brain regions and edges signify the statistical interdependence of CBF between nodes. The brain was divided using the automated anatomical labeling (AAL) template, which includes 90 cerebral and 26 cerebellar regions, with each region defined as a node (17). The mean CBF value of each node was extracted for each subject, and linear regression was applied at each node to account for the effects of age and gender. Pearson correlation coefficients between the residuals of each node pair across all subjects were used to define edges (18). This process generated a 116 × 116 correlation matrix for each group. This weighted network leverages the strength of CBF coupling to better characterize network topology compared to a binary network. A sparsity threshold, defined as the ratio of the existing edges to the maximum possible number of edges, was applied to the correlation matrices. This minimized the impact of intergroup differences in overall correlation strength and ensured that all networks contained the same number of edges. Since sparsity threshold selection can influence network analysis results, network properties were calculated across a broad range of sparsity thresholds. These thresholds were selected based on two criteria: (1) the average node degree (the number of connections linked to a node) across all networks exceeded 2log(N) (19), where *N* is the number of nodes (*N* = 116); and (2) the resulting network exhibited sparse, distinct properties compared to a degree-matched random network. Based on these criteria, the sparsity levels ranged from 0.09 to 0.30, with a step size of 0.01.

At each sparsity threshold, we calculated several key global parameters, including the clustering coefficient Cp (the average of the clustering coefficients of all nodes in the network, reflecting the connection state of the entire network), characteristic path length Lp (measures the extent of network long-distance connections), normalized clustering coefficient *γ* (a ratio of the clustering coefficient between real and 100 random networks, which quantifies the local interconnectivity of a network), normalized characteristic path length *λ*(a ratio of the characteristic path length between real and 100 random networks, which quantifies the overall routing efficiency of a network), small-worldness *σ* = *γ* / λ(measures the small-worldness of a network), local efficiency Eloc (measures of the fault tolerance of the network), and global efficiency Eglob (measures the ability of parallel information transmission over the network). Importantly, Lp was calculated using the harmonic mean distance between all node pairs to address issues related to potentially disconnected network components.

### Statistical analysis

Demographic and clinical characteristics were compared between groups using the chi-square test or Fisher’s exact test for categorical variables and either Student t-test or the Mann–Whitney U test for continuous variables. A multiple linear regression model was used to assess the association between intravenous thrombolysis and cognitive function scores. The first model was adjusted for age, sex, years of education, and treatment modality. The second model further adjusted for NIHSS, TOAST classification, infarct location, hypertension, diabetes, dyslipidemia, coronary artery disease, atrial fibrillation, and current smoking. The CBF covariance network analysis was performed using nonparametric permutation tests with 1,000 permutations to assess statistical significance, as network measures were obtained at the group level. For each measure, we first calculated the real intergroup difference. Subjects were then randomly reassigned to groups, maintaining the original group sizes, and the network construction and small-world property calculations were repeated. We counted how often the differences in the permuted network measure exceeded the real difference (14). The *p*-value for each measure was derived by dividing the number of such occurrences by the total permutations. Statistical significance was set at *p* < 0.05 for each test.

### Validation analysis

To evaluate the impact of network type on our findings, we repeated the same analyses on the binary network as we did on the weighted network.

## Results

### Demographic and clinical data of subjects

This study included 50 healthy controls and 94 first-time mild stroke patients (44 IVT and 50 non-IVT). The two groups were matched for gender (27 males and 67 females for the patient group; 22 males and 28 females for the control group; *p* = 0.065) and age (60.9 ± 9.0 years for the patient group; 62.9 ± 2.8 years for the control group; *p* = 0.051). However, the MoCA scores were significantly lower in the mild stroke patients (*p* < 0.001). The detailed demographic and clinical data are presented in Table 2.

**Table 2:** Differences in clinical characteristics and cognitive results (*z*-scores) between mild stroke and control.

Baseline characteristics	All	Mild stroke	HC	*p*-values
Age	61.6 ± 7.5	60.9 ± 9.0	62.9 ± 2.8	0.051
Male sex-no. (%)	95 (65.9)	67 (71.3)	28 (56.0)	0.065
Education, y	9 (5.3–11)	8 (6–11)	11 (5–11)	0.058
Cognitive scores				
MoCA		–0.25 ± 1.05	0.46 ± 0.69	<0.001

All continuous variables are presented as the mean ± SD. SD, standard deviation; HC, healthy control. *P* < 0.05 was defined as being statistically significant.

Among the 94 patients with a first mild stroke, there were no significant differences in age, sex, education, medical history, presumed stroke cause, or location of cerebral infarction between the IVT and non-IVT groups (Table 3). Notably, patients who received IVT had higher NIHSS scores. Significant differences between the IVT and no-IVT groups were found in TMT-B (*p* = 0.043), CDT (*p* < 0.001), and VFT (*p* = 0.033). However, there were no significant differences in MoCA scores, HVLT, or TMT-A.

**Table 3:** Clinical characteristics and cognitive results (*z*-scores) in the full analysis set.

Baseline characteristics	IVT	Non-IVT	*P*-values
Age	60.5 ± 9.9	60.5 ± 8.7	0.974
Male sex-no. (%)	31 (70.5)	36 (72.0)	0.869
Education, y	8 (5.3–11)	8.5 (5.8–11)	0.731
NIHSS score	3 (3–4)	2 (1–3)	<0.001
Presumed stroke cause			0.546
Large vessel disease	16	15	
Small vessel disease	27	34	
Cardio embolic disease	1	0	
Undetermined cause	0	1	
Topography -no. (%)^a^			0.924
Right hemisphere	16	17	
Left hemisphere	22	27	
Cerebellum/brainstem	6	6	
Medical history			
Hypertension	39	40	0.277
Diabetes	9	13	0.628
Dyslipidemia	26	20	0.098
Smoker	20	27	0.408
Coronary artery disease/prior MI	3	2	0.662
Atrial fibrillation/flutter	2	0	0.216
Cognitive scores			
MoCA	0.20 ± 0.82	−0.17 ± 1.11	0.072
TMT-A	0.21 ± 1.10	−0.19 ± 0.87	0.054
TMT-B	0.23 ± 1.12	−0.20 ± 0.84	0.043
HVLT-R total recall	0.20 ± 1.04	−0.18 ± 0.94	0.070
HVLT-R delayed recall	0.20 ± 1.01	−0.18 ± 0.97	0.068
HVLT-R recog	−0.03 ± 1.08	0.02 ± 0.93	0.815
CDT	0.35 ± 0.79	−0.31 ± 1.07	0.001
VFT	0.23 ± 0.90	−0.21 ± 1.05	0.033

MoCA, montreal cognitive assessment; HVLT, hopkins verbal learning test; TMT, trail making test; CDT, clock drawing test; VFT, verbal fluency test; The values are median ± SD or median (IQR). SD, standard deviation; IQR, interquartile range.

^aThe location of the largest infarcted lesion. P < 0.05 was defined as being statistically significant.^

### The association between IVT and cognitive scale scores

We analyzed the association between IVT and cognitive scale scores with 2 multivariable models. In Model 1 adjusted for covariates including age, sex, and education years, IVT demonstrated significant associations with MoCA, HVLT-R total recall, HVLT-R delayed recall, CDT, VFT, TMT-A, and TMT-B scores. Furthermore, these associations remained significant after further adjustment for NIHSS, TOAST classification, hypertension, diabetes, dyslipidemia, coronary artery disease, atrial fibrillation, and current smoking status (Table 4).

**Table 4:** Association of IVT with cognitive scale scores (non-IVT vs. IVT).

Cognitive scores, *β* (95%CI)	Model 1	Model 2
MoCA	2.50 (0.63 to 4.36) **	2.89 (0.64 to 5.13) *
TMT-A	−15.89 (−29.03 to −2.76) *	−16.90 (−32.89 to −0.90) *
TMT-B	−30.51 (−60.81 to −0.21) *	−43.27 (−78.78 to −7.76) *
HVLT-R total recall	2.33 (0.41 to 4.26) *	3.57 (1.36 to 5.78) **
HVLT-R delayed recall	1.13 (0.18 to 2.08) *	1.53 (0.43 to 2.63) **
HVLT-R recog	−0.03 (−1.10 to 1.04)	0.66 (−0.60 to 1.91)
CDT	2.55 (1.14 to 3.95) **	3.01 (1.33 to 4.68) **
VFT	5.31 (1.43 to 9.18) **	7.09 (2.40 to 11.79) **

Model 1: adjusted for age, gender and education years. Model 2: adjusted for all age, gender, education years, NIHSS, TOAST classification, infarct location, hypertension, diabetes, dyslipidemia, coronary artery disease, atrial fibrillation, current smoking. *Means a *p* value < 0.05; **Means a *p* value < 0.01.

### Differences in cerebral blood flow

**Figure 1:**
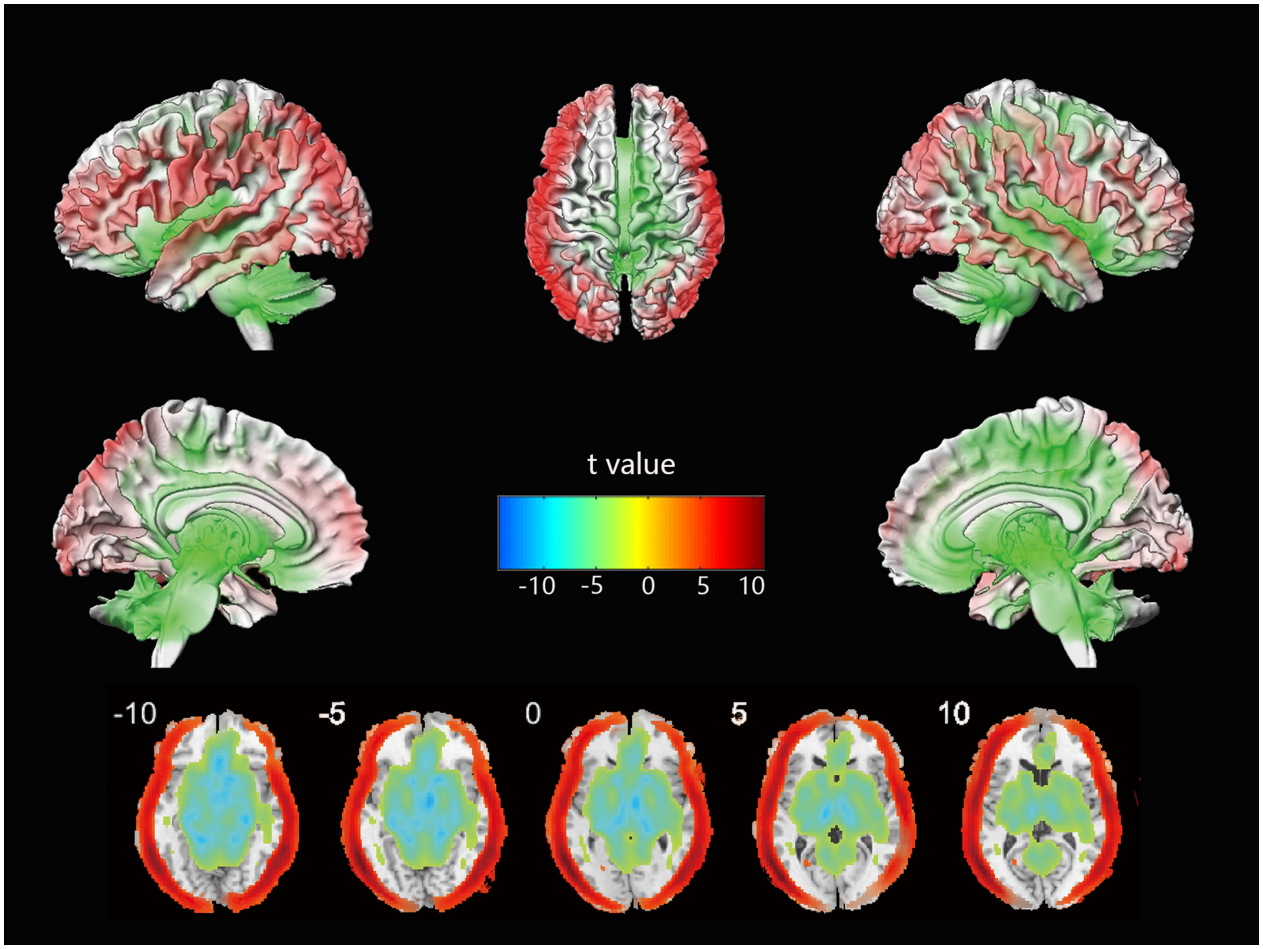
The voxel-wise CBF differences between mild stroke patients and healthy controls.

The voxel-wise CBF differences between mild stroke patients and healthy controls are shown in Figure 1. Cortical perfusion in the healthy control group was better than in mild stroke patients, while subcortical perfusion showed the opposite trend. In a further comparison of treatment methods for mild stroke, no significant differences in cortical or subcortical perfusion were observed between the IVT and no-IVT groups.

### Functional covariance network

**Figure 2:**
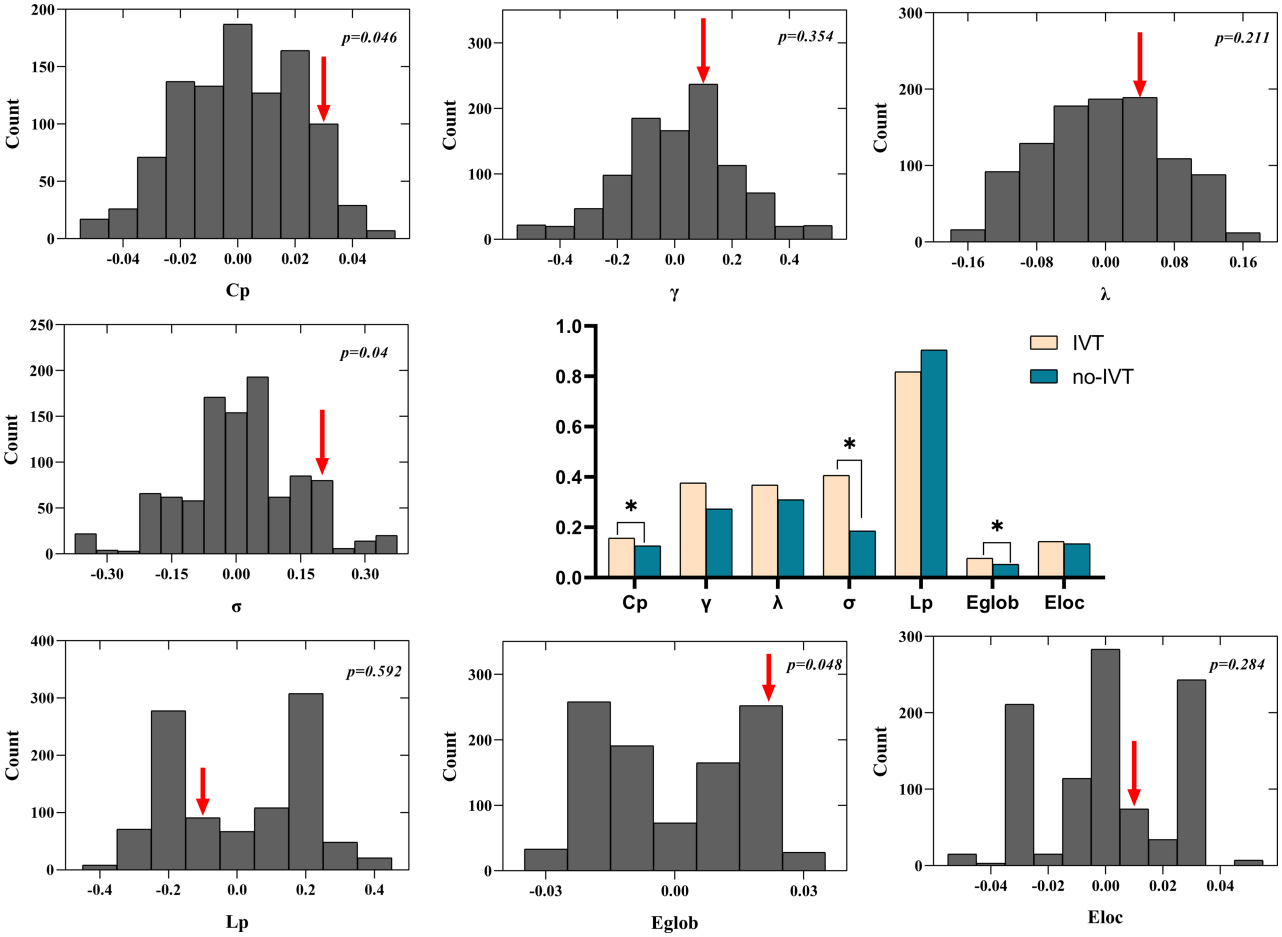
Global topological differences in CBF covariance network between IVT patients and non-IVT patients. The black stars in bar plots denote statistically significant differences between the two groups (permutation test, *p* < 0.05). The histogram plots around the bar plots are null distributions of permutation tests of global network measures and the real measures are marked with red arrows. IVT, intravenous thrombolysis; non-IVT, non-intravenous thrombolysis.

There were significant differences in several parameters of the CBF covariance network between IVT and no-IVT patients (Figure 2). Compared to no-IVT patients, IVT patients exhibited significantly increased characteristic path length (0.157 vs. 0.126, *p* = 0.046), Sigma (0.406 vs. 0.185, *p* = 0.04), and Eglob (0.077 vs. 0.053, *p* = 0.048). We also observed that our primary results were consistent in the validation analyses of the binary network (Supplementary Figure S1).

## Discussion

In this study, we found that IVT was associated with early cognitive benefits. Utilizing graph theory, we further explored its effects on the cerebral blood flow (CBF) covariance network in mild ischemic stroke patients. Our findings demonstrated that: (1) Patients exhibited CBF abnormalities characteristic of mild ischemic stroke (2) IVT attenuated damage to the CBF covariance network. Specifically, patients treated with IVT demonstrated higher clustering coefficients, small-worldness, and global efficiency compared to those who did not receive this treatment, suggesting less severe damage to their brain networks.

The longitudinal trajectory of post-stroke cognition exhibits dynamic fluctuation, where baseline cognitive status serves as critical predictor for long-term impairment development (20). Our findings demonstrate that mild stroke induces multi-domain cognitive impairments, characterized by heterogeneous lesion-specific deficits superimposed on diffuse vascular injury, which are not entirely consistent with the executive/attentional/visuospatial deficits typical of vascular cognitive impairment. Since our healthy control individuals were from a different study that used specific cognitive domain scales (memory, visuospatial ability, language ability) which were not consistent with this study, we used the overall cognitive assessment scale MoCA for comparison. Fortunately, due to MoCA’s good sensitivity, we were able to obtain results indicating cognitive impairment in mild stroke patients. As a first-line treatment for acute ischemic stroke, IVT is effective at dissolving blood clots and restoring cerebral blood flow, which is essential for maintaining cognitive function. Despite limited research on its effects on cognitive outcomes in mild stroke populations, our results supported the protective role of IVT on cognitive function (7, 16). Notably, these findings persisted despite the initial IVT recipients exhibiting higher NIHSS scores.

Decreased cerebral blood flow is linked to cognitive decline, often preceding the onset of vascular cognitive impairment symptoms. Thus, measuring CBF can aid in the early identification of vascular cognitive impairment risk. Previous studies indicate that higher overall brain blood flow, as well as subcortical flow and blood flow in the frontal and temporal lobes, correlates with better cognitive function (21–22). However, when comparing thrombolysis patients to the non-thrombolysis group, we did not observe significant advantages in cerebral blood flow.

Research on brain networks is crucial for understanding cognition. Normal cognitive function depends on an intact, efficient brain network, while pathological conditions can disrupt network connectivity. Graph theory is a mathematical tool for analyzing and quantifying brain networks. It provides a simplified model of the brain connectome, representing it as a collection of nodes and edges that reflect the overall network structure (23). By abstracting the complex network structure of the brain into simple geometric representations, it is possible to calculate parameters that describe specific topological properties of the network, allowing for the study of both individual nodes and the entire network (23, 24). Mild stroke patients with cognitive impairment show lower clustering coefficients and global efficiency, along with higher characteristic path lengths in their structural brain networks (24, 25). Given our finding of significant cerebral blood flow abnormalities in mild stroke, we believe it is likely that an abnormal CBF covariance network based on cerebral blood flow can be established. This method has proved its reliability in many studies (13, 14). Although the locations of cerebral infarction lesions can significantly influence comparisons of brain network properties, our study focuses on global attributes. We observed that patients in the IVT group showed superior network properties in the CBF covariance network, particularly in terms of cluttering coefficients, small-worldness, and global efficiency, compared to patients in the non-IVT group. The underlying mechanisms where IVT protects the brain network are not yet clear. We speculate that intravenous thrombolysis rapidly restores the blood supply to the brain, reduces further death of neuronal cells, mitigates the degree of blood–brain barrier disruption, and thus controls the progression of inflammatory responses.

Although weighted edges provide a more precise representation of the brain network compared to binary ones, they might be more susceptible to noise. Our findings revealed analogous disparities in the topological characteristics of binary networks between the two groups, indicating that our results are robust regardless of the network type analyzed.

Previous studies have shown that more robust CBF covariance networks correlate with higher cognitive levels (26, 27). Our study found that the global properties of the CBF covariance network improve after intravenous thrombolysis; however, in cognitive assessments, the thrombolysis group demonstrated advantages only in specific cognitive domains. Post-stroke cognitive functions are not static and may follow different trajectories as the disease progresses (28). We speculate that the CBF covariance network may provide early evidence of impairment in other cognitive domains, which will require further longitudinal studies. Some longitudinal studies have indicated that network restructuring may occur as the duration of illness extends (29). Therefore, we recognize the necessity for future longitudinal follow-up studies to further explore the connection between brain networks and cognition, particularly the significance of early CBF covariance networks for the occurrence and persistence of future PSCI.

Our study has some strengths. It is one of the few studies focusing on cognitive impairment following intravenous thrombolysis for mild stroke, particularly including relevant MRI indicators, which avoids the simplistic assessment of a single cognitive scale and enables more objective and quantitative research. Based on the mechanism by which intravenous thrombolysis can restore cerebral blood flow, we analyzed the differences in brain networks from the unique perspective of CBF covariance networks. However, our study still has its limitations. First, the sample size of our study is relatively small. In the future, conducting randomized controlled trials and increasing the sample size would significantly enhance the quality of the research. Second, since all covariance networks can only be constructed at the group level, we are unable to investigate the relationships between topological properties and individual cognition. Lastly, the impairment of cognitive function after stroke requires long-term follow-up. Exploring the abnormalities in early CBF covariance networks and their association with the onset and progression of PSCI will be the focus of our next steps.

## Conclusion

Our study suggested that IVT may alleviate cognitive impairment after mild ischemic stroke. Improvement in the brain CBF network properties may be the underlying mechanism.

## Data Availability

The original contributions presented in the study are included in the article/Supplementary material, further inquiries can be directed to the corresponding author.
