# Supplementary material for: Cognitive impairment after intravenous thrombolysis in mild stroke: assessment of cerebral blood flow covariance network
Source: Front Neurol. 2025 Mar 7;16:1513182. doi: 10.3389/fneur.2025.1513182 (PMC11925760; doi:10.3389/fneur.2025.1513182)
Supplement: Supplementary file 1 [file Table_1.DOCX]

Cognitive impairment after intravenous thrombolysis in mild stroke： Assessment of Cerebral Blood Flow Covariance Network

**Kefu Mei^1,2^,Feng Li^3^,Zhiming Kang^1^,Dong Sun^1^,Xuefei Luo^4^,Shiyuan Tian^2^,Lei Zhang^2^,Junjian Zhang^1^**

^1^Department of Neurology, Zhongnan Hospital of Wuhan University, Wuhan, China

^2^Department of Neurology, Xiangyang Central Hospital, Affiliated Hospital of Hubei University of Arts and Science, Xiangyang, China.

^3^Department of Radiology, Xiangyang Central Hospital, Affiliated Hospital of Hubei University of Arts and Science, Xiangyang, China.

^4^Xiangyang Polytechnic, Xiangyang, China.


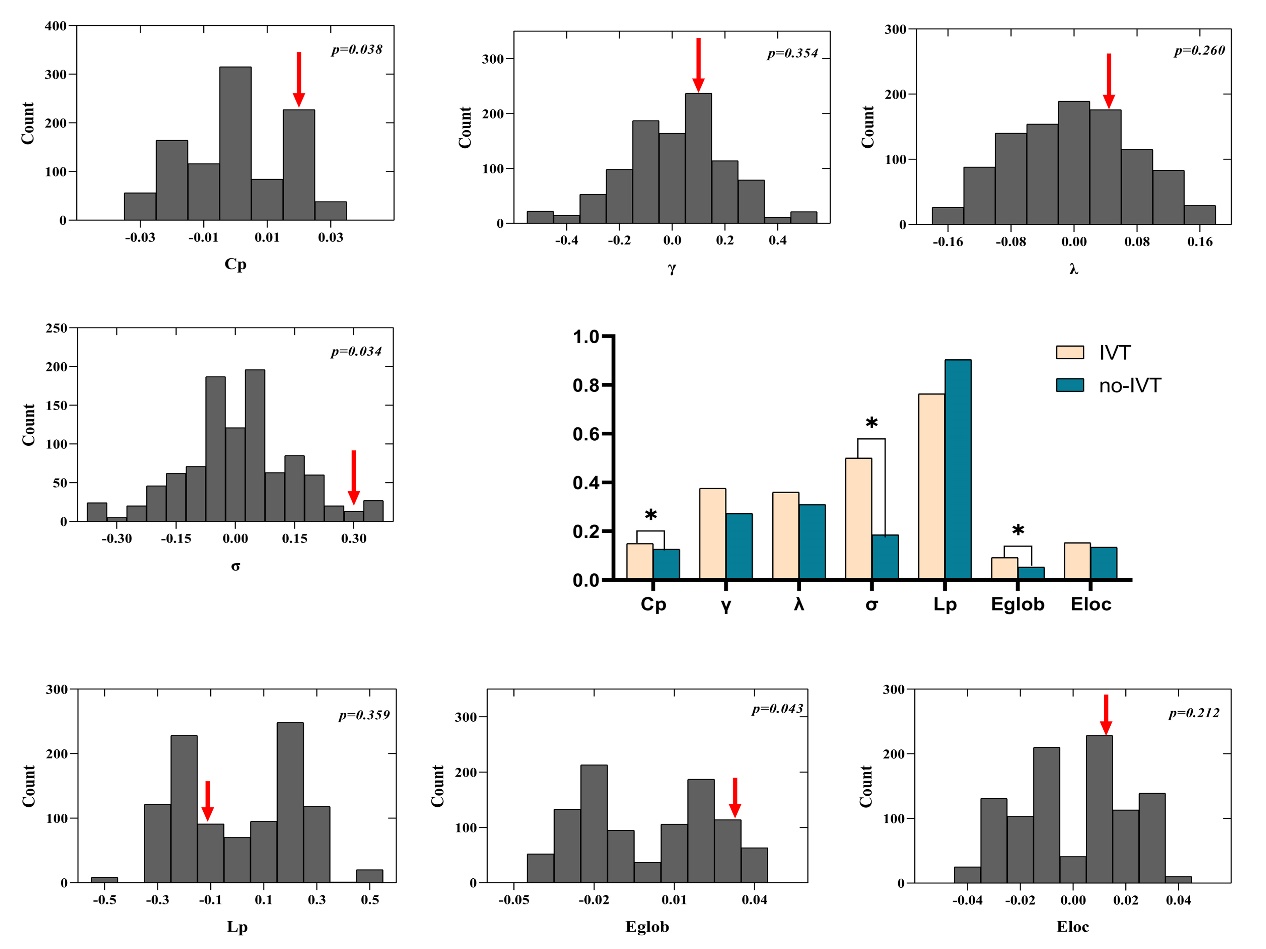


**Figure S1 | Global topological differences in CBF binary covariance network between IVT patients and non-IVT patients. The black stars in bar plots denote statistically significant differences between the two groups (permutation test, p <0.05). The histogram plots around the bar plots are null distributions of permutation tests of global network measures and the real measures are marked with red arrows. IVT, intravenous thrombolysis; non-IVT, non-intravenous thrombolysis.**
